# Motion Smoothness Metrics for Cannulation Skill Assessment: What Factors Matter?

**DOI:** 10.3389/frobt.2021.625003

**Published:** 2021-04-16

**Authors:** Simar Singh, Joe Bible, Zhanhe Liu, Ziyang Zhang, Ravikiran Singapogu

**Affiliations:** ^1^ Department of Bioengineering, Clemson University, Clemson, SC, United States; ^2^ Department of Mathematical and Statistical Sciences, Clemson University, Clemson, SC, United States

**Keywords:** motion smoothness, cannulation, medical training simulator, log dimensionless jerk, spectral arc length, skill metrics

## Abstract

Medical training simulators have the potential to provide remote and automated assessment of skill vital for medical training. Consequently, there is a need to develop “smart” training devices with robust metrics that can quantify clinical skills for effective training and self-assessment. Recently, metrics that quantify motion smoothness such as log dimensionless jerk (*LDLJ*) and spectral arc length (*SPARC*) are increasingly being applied in medical simulators. However, two key questions remain about the efficacy of such metrics: how do these metrics relate to clinical skill, and how to best compute these metrics from sensor data and relate them with similar metrics? This study addresses these questions in the context of hemodialysis cannulation by enrolling 52 clinicians who performed cannulation in a simulated arteriovenous (AV) fistula. For clinical skill, results demonstrate that the objective outcome metric flash ratio (**FR**), developed to measure the quality of task completion, outperformed traditional skill indicator metrics (years of experience and global rating sheet scores). For computing motion smoothness metrics for skill assessment, we observed that the lowest amount of smoothing could result in unreliable metrics. Furthermore, the relative efficacy of motion smoothness metrics when compared with other process metrics in correlating with skill was similar for **FR**, the most accurate measure of skill. These results provide guidance for the computation and use of motion-based metrics for clinical skill assessment, including utilizing objective outcome metrics as ideal measures for quantifying skill.

## 1 Introduction

The health of populations is directly related to a well-trained healthcare workforce; therefore, attention must be given to training our clinical professionals efficiently and safely. There is mounting evidence that training not only results in better clinical outcomes but also decreases costs and procedural times [Farnworth et al. (2001)]. To facilitate training, simulators have been gaining increasing popularity in medical education due to their ability to quantify skill in a simulated environment while providing feedback on performance. Further, simulators often enable self-paced learning *via* metrics that track the performance of trainees over time. In recent years, simulator training has demonstrated positive results in arthroscopy, laparoscopy, and endovascular surgery [Rosen (2008), Hafford et al. (2013), Duran et al. (2015), Goyal et al. (2016)]. Many simulators used in these studies provided skill assessment objectively (i.e., through sensor-based metrics) and not the “subjective” assessment of a trainer. Simulator-based training does not require live animal models or expert trainers and can be done remotely. Also, studies have brought to attention the fact that novice medical students can tend to overestimate their ability [MacDonald et al. (2003)]. Thus, tools must be provided that accurately and reliably help assess a trainee’s skill and move students towards proficiency.

In the last few decades, numerous studies have reported on various simulator-based metrics used in their simulators to distinguish the skilled performance of a simulated medical procedure, among which time (*T*) and path length (*PL*), the length traversed by a medical instrument during task performance, are frequently used. For instance, training on a simulator has yielded significant differences in completion time after training on the simulator [Judkins et al. (2009)] and in completion time after 1 h of training in a clinical environment [Pedowitz et al. (2002)]. With current simulators featuring state-of-the-art sensors, numerous types of data can be recorded and used for computing metrics. In a recent study using an arthroscopic “box” simulator, time and errors were used to successfully differentiate medical students from novices in two tasks [Braman et al. (2015)], whereas the Fundamentals of Arthroscopic Surgery Training (FAST) saw similar results with the same metrics across four tasks. *PL* is used in various simulators to determine significant differences among experts and nonexperts [Pedowitz et al. (2002), Jacobsen et al. (2015)]. Metrics such as *PL* and *T*, while useful for skill assessment, are somewhat rudimentary measures of skill since they focus on basic aspects of clinical skill.

Dexterity has long been regarded as one of the hallmarks of a good clinician since many medical procedures involve precise and deft handling of instruments. Towards quantifying dexterity, motion smoothness metrics have been recently applied in medical simulators. The primary facet that these metrics capture is the “smoothness” of a motion while performing the task, leading to an understanding of in-the-process task performance. Since they were first proposed about two decades ago with rudimentary metrics such as the number of peaks in the velocity profile (*Pks*) or various jerk formulations, motion smoothness metrics have evolved in their robustness for skill assessment. Currently, two motion smoothness metrics, log dimensionless jerk (*LDLJ*) and spectral arc length (*SPARC*), are being explored for quantifying medical skills. One difficulty encountered by researchers seeking to incorporate these metrics is their dependence on computing derivatives from often noisy sensor data. Depending on how derivatives are computed, noise present in the raw sensor data may be heavily magnified with each order of derivative. To remedy erratic data, smoothing is often performed on sensor data. However, “oversmoothing” the data can filter out important motion features that may be necessary for skill assessment. Therefore, it is vital to examine the effect of smoothing parameters on the computation of motion smoothness metrics and their relative efficacy in quantifying skill in comparison with “traditional” metrics (e.g., *T* and *PL*). This work seeks to contribute towards a greater understanding of these two issues.

The context of the current work is evaluating the skill of cannulating a simulated arteriovenous (AV) fistula, a vascular access into the patient’s bloodstream, for hemodialysis. This is an operation that is done in dialysis clinics around the world by nurses or patient care technicians tens of times each week. Though this is a relatively simple procedure involving inserting a 14-17 gauge needle into a blood vessel, the quality of cannulation is extremely important for patient health as multiple failures and attempts can lead to several complications such as hematoma, infection, and aneurysm formation that could lead to eventual death [Van Loon et al. (2009)]. One of the main reasons for miscannulation is infiltration: where the clinician punctures through the AV fistula and causes blood to leak out [Brouwer (2011)]. A frequent result of severe miscannulation is a need for surgical treatment of the vascular access, presenting increased risk to the patient. Hospital readmissions can lead to exposure of patients to pathogens that present a greater risk of mortality because of patient comorbidities. Our team has created a simulator for practicing cannulation for hemodialysis in a safe environment with the ability to provide objective metrics based on motion, force, and time data [Zhang et al. (2019), Liu et al. (2020a), Liu et al. (2020b)]. In this study, data from nurses and patient care technicians with various degrees of experience are analyzed for performance characteristics on the cannulation simulator, which yields insights into what constitutes skilled cannulation for hemodialysis.

In addition to systematically quantifying the strength of motion smoothness metrics for cannulation skill, another key contribution of this study is the use of an objective metric to measure the outcome of cannulation. Our simulator features relevant hardware and software to track whether the needle is inserted into the fistula accurately (i.e., for blood withdrawal) and if, during the process, any degree of infiltration was encountered [Liu et al. (2020b)]. By using an outcome metric that is objective regarding the success of the task, we reduce or eliminate the need for other, more “traditional” measures used as surrogates for skill that may inaccurately appraise skill. Two such commonly used measures are years of experience (or the number of cases performed, e.g., in the case of surgeons) and the rating of experts regarding the performance of a task/procedure. Both of these metrics have inherent limitations. In the case of clinical experience, when this measure is used as a surrogate for skill, the implicit assumption is that greater experience results in improved skill; however, this may not be the case as some studies suggest [Hung et al. (2019), Liu et al. (2021)]. By classifying expertise based on clinical experience, we may be inaccurately estimating skill. The other metric commonly used to measure skill is a Likert-scale rating assessed by an expert who witnesses the task being performed. While there is value to these evaluations by experts, the limitations of such a method include the inherent subjectivity of raters and the fact that raters often give one “global” rating for the whole task. In this work, we not only introduce a new outcome metric but also provide insights into how this metric compares with both traditional measures used to gauge skill.

## 2 Motion Smoothness

In various specialized as well as everyday tasks, precise and controlled motion is required for successful execution. Some domains where this is particularly the case are sports, surgery, and rehabilitation. To measure skilled performance in these cases, one cannot simply rely on metrics like time to completion or economy of motion. While these have been proven to be useful to assess skill in many cases, these metrics are not designed to quantify the smoothness (or lack thereof) of movement since they do not measure in-the-process motion. As an example, *PL*, a commonly used metric, only measures the total length of the motion traversed during a motion; it does not quantify how smooth that motion was. Some studies demonstrate moderate utility for *Pks* to examine smoothness of motion, with limited usage due to lack of generalizability and robustness [Balasubramanian et al. (2012), Balasubramanian et al. (2015), Estrada et al. (2016), Gulde and Hermsdörfer (2018a)]. In the following subsections, we detail more advanced formulations of motion smoothness metrics that are based on higher-order derivatives of position data. It should be noted that these metrics have evolved in their complexity and applicability since they were first devised for use in rehabilitative treatments [Flash and Hogan (1985)]. We discuss the particular strengths and weaknesses of each metric along with their use cases.

### 2.1 Jerk

In an ideal, smooth motion, acceleration would not have any discontinuities, as could be determined by the derivative of acceleration, jerk. This notion has served as the key idea for quantifying motion smoothness. However, computing “pure” jerk is too inconsistent to be used as a measure of motion smoothness [Hogan and Sternad (2009)]. For example, some studies did not detect significant differences in jerk values among motor movements in unhealthy and healthy patients [Goldvasser et al. (2001), Wininger et al. (2009)], while other studies demonstrate otherwise [Teulings et al. (1997), Smith et al. (2000), Rohrer et al. (2002)]. From these studies, it was observed that jerk should be normalized as it depends heavily on movement duration and range of motion and that minimizing jerk is essential for smooth motion quantification.

Flash and Hogan (1985) proposed minimizing the cost function of jerk by squaring and integrating the value as a viable metric for motion smoothness This measure, known as integrated square jerk, and others based on this measure were used in several studies to quantify smooth motion [Smith et al. (2000), Goldvasser et al. (2001), Rohrer et al. (2002), Wininger et al. (2009)]. Eventually, these metrics were termed dimensioned jerk metrics since they rely on the duration and amplitude of movement. Later, Hogan and Sternad (2009) created a new metric that eliminated such reliance. This metric, known as dimensionless jerk, accounted for measuring the intermittency in motion regardless of its duration or amplitude. Intermittency in a discrete motion can arise from the lack of controlled movement, characterized by a period of deceleration preceding a point of acceleration, or can be due to finite periods of no motion from uncertainty. Balasubramanian and colleagues noted that for a motion smoothness metric to be valid, it must have the following features: it must be dimensionless, monotonically responsive to motion, sensitive to changes in movement, and feasible for computation [Balasubramanian et al. (2012)].

Dimensionless jerk (*DLJ*) has been used in several recent studies to assess clinical skills. In one recent study, participants performed a pegboard placement task, with *DLJ* able to differentiate among surgeons and nonsurgeons as well as among the tasks tested [Ghasemloonia et al. (2017)]. Further, *DLJ* was also employed in a simulated shoulder arthroscopy test, where significant differences between experts and novices were evidenced in *DLJ* values [Kholinne et al. (2018)]. This metric has also been used in a Fundamentals of Endovascular Skills (FEVS) trainer [Estrada et al. (2016), O’Malley et al. (2019)], where it differentiated between novice and expert skill. One notable limitation, however, is that the values for *DLJ* vary widely, differing by the thousands between users in some cases. To eliminate this wide variability, the use of the natural log of dimensionless jerk (*LDLJ*) has been proposed [Balasubramanian et al. (2012), Balasubramanian et al. (2015), Gulde and Hermsdörfer (2018b), Melendez-Calderon et al. (2020)]. Studies report more robust measurements and better sensitivity of this metric to the physiological hand motion range. Balasubramanian and others emphasize that *LDLJ* is often affected by a signal noise since calculating jerk involves computing the third derivative [Balasubramanian et al. (2012)]. This observation leads to one of the research questions addressed in this study.

### 2.2 Spectral Arc Length

In 2012, a new metric to quantify motion smoothness that was more robust to noise was formulated, known as spectral arc length (*SPARC*) [Balasubramanian et al. (2012), Balasubramanian et al. (2015)]. The metric is derived from the arc length of the amplitude of the frequency-normalized Fourier magnitude spectrum of the velocity profile. This metric is based on the observation that smooth hand movements will yield small magnitudes of low-frequency profiles, whereas “unsmooth” movements will yield large magnitudes of different higher-frequency profiles. The larger the magnitudes of different frequency movements are, the more the arc length of the profile increases. This idea is analogous to minimizing the cost function of jerk. Since this metric relies on analyzing motion *via* the frequency domain, it is more robust to noise and sensitive to changes in smaller movements [Balasubramanian et al. (2012)]. *SPARC* is being increasingly used to measure skilled or smooth motion, including in the previously mentioned FEVS studies [Duran et al. (2015), O’Malley et al. (2019), Belvroy et al. (2020)], in which it consistently demonstrates strong correlations to skill between experts and novices. In this study, we systematically compare the efficacy of both *SPARC* and *LDLJ* for quantifying cannulation skill—another clinical skill that requires smooth motion. A summarized list of metrics is shown in Table 1.

**TABLE 1:** List of commonly used process metrics for evaluating skilled motion. Several previous studies have used these metrics for various clinical applications.

Metric	Equation
Time	$t_{2}-t_{1}$
Path length	$\overset{t_{2}}{\underset{t_{1}}{\int }}\frac{dX}{dt}$
Peaks in the velocity profile	$\Sigma v_{\max ima}$
Integrated square jerk	$-\overset{t_{2}}{\underset{t_{1}}{\int }}{\left(\frac{d^{3}x}{dt^{3}}\right)}^{2}$
Dimensionless jerk	$-\frac{T^{5}}{PL^{2}}\overset{t_{2}}{\underset{t_{1}}{\int }}{\left(\frac{d^{3}x}{dt^{3}}\right)}^{2}$
Log dimensionless jerk	$-\ln\left|\frac{T^{5}}{PL^{2}}\overset{t_{2}}{\underset{t_{1}}{\int }}{\left(\frac{d^{3}x}{dt^{3}}\right)}^{2}\right|$
Spectral arc length	$-\int_{0}^{\omega_{c}}{\left[{\left(\frac{1}{\omega_{c}}\right)}^{2}+\left(\frac{d\hat{V}\left(\omega \right)}{d\omega }\right)\right]}^{\frac{1}{2}}d\omega ;$
	$\hat{V}\left(\omega \right)=\frac{V\left(\omega \right)}{V\left(0\right)}$

## 3 Motivation of Study

In this study, we examine three research questions relevant to the use of motion smoothness metrics for clinical skill assessment and training. We present each question, followed by a rationale motivating the question.

To summarize this section, the three research questions addressed in this work are as follows:

• Which skill indicator metric best accounts for dexterity measures?

We define two types of metrics in this study: skill indicator metrics and process metrics. Skill indicator metrics [e.g., global rating sheet (**GRS**)] seek to appraise the skill of clinicians while process metrics (e.g., *LDLJ*) quantify certain characteristics of task performance. To better differentiate between the two, process metrics are abbreviated with an italicized font, whereas skill indicator metrics are abbreviated with a bold font. We introduce a novel metric for objectively assessing skill by determining the degree of success in cannulation task outcome. However, of the three skill indicator metrics used in this study—**GRS**, clinical experience, and task outcome—which one best characterizes clinical skill? The answer to this question has important implications in the way clinical skill is classified in studies. Further, many metrics are task-specific by definition. That is, they are effective to the degree that they accurately reflect the task. Motion smoothness measures are known to be task-specific: motion smoothness values indicative of skill are not equivalent across different tasks [Balasubramanian et al. (2012)]. Thus, we examine which of the three skill indicator metrics is best correlated with the suite of process measures in this study. Another aspect of our study about the way skill is classified needs to be mentioned here. In many studies, skill is binarized as either expert or novice, greatly simplifying the notion of skill. Our work denotes a skill as a continuum wherein each skill indicator metric can take on a range of values, thus enabling a fine-grained classification of skill.

• Does the degree of smoothing of sensor data significantly affect the computation of motion smoothness metrics?

**FIGURE 1:**
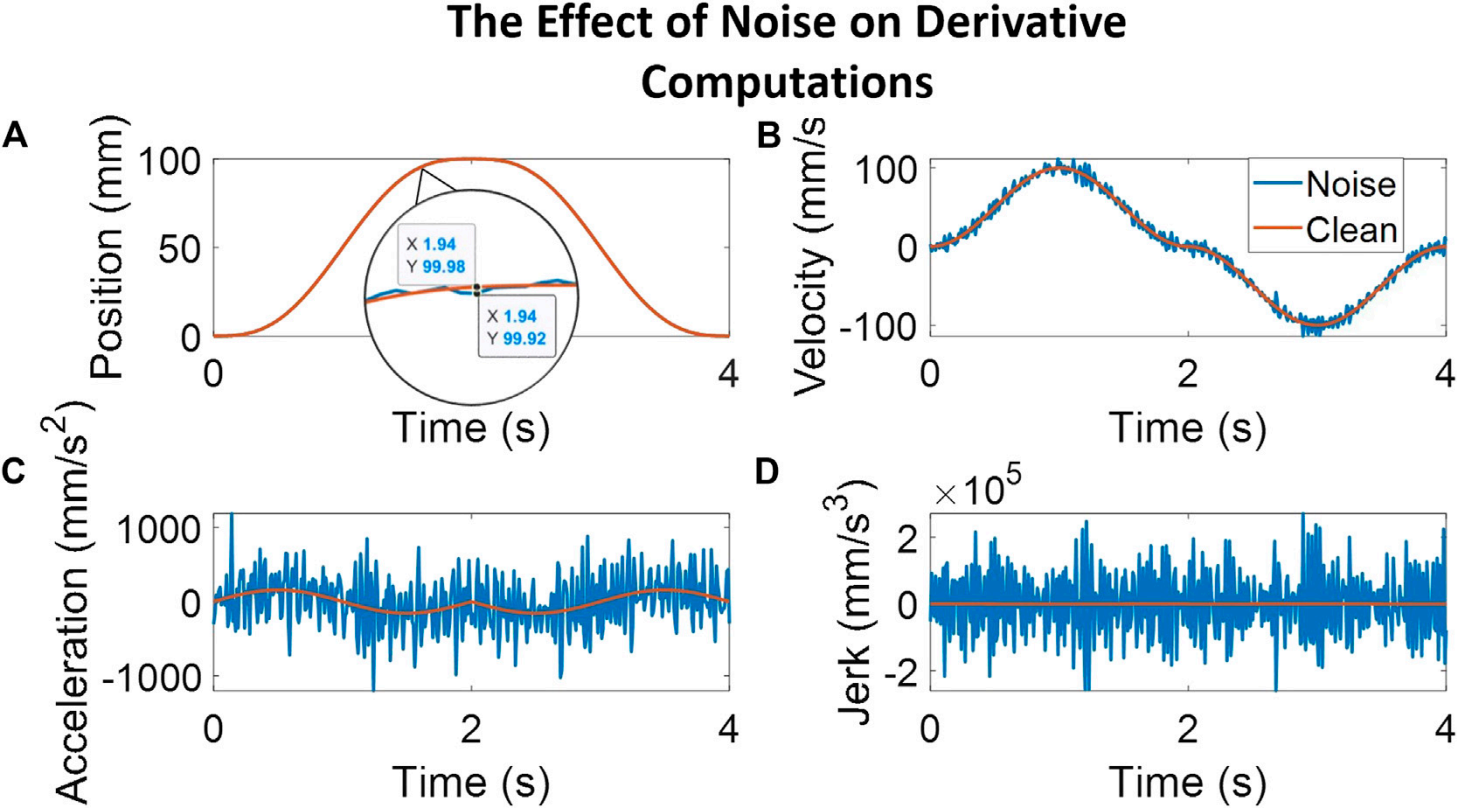
The plots here follow a simulated movement profile and a corresponding movement profile with noise added to illustrate the effect of noise on computing higher-order derivatives. The noise adds peaks averaging 0.06 mm, as seen in the magnified position plot. Subplot **(A)** shows the position profile with a magnified view of the clean signal overlaid with the noisy signal. The other subplots illustrate the results of computing derivative estimates for velocity [subplot **(B)**], acceleration [subplot **(C)**], and jerk [subplot **(D)**] at each step. As can be noted, even a small amount of noise in the position profile causes a relatively noisy jerk profile, calculated here through a third-order Savitzky–Golay filter with a window span of five.

Studies that involve assessing or training clinical skills on simulators most often use data from sensors to provide feedback to trainees. To extract motion smoothness metrics, however, the inherent noise in sensor data poses a problem while computing higher-order derivatives. As such, it is important to understand the role of data smoothing—the type and the degree of smoothing used—on the computation of metrics. To our knowledge, no study thus far has presented the effect of the degree of smoothing for measuring clinical skill. A related study demonstrated that, for computing *SPARC* and *PL*, optimal filtering was obtained for a specific window span range and for a specific type of filter [Gulde and Hermsdörfer (2018a)]. This study, however, did not include computation of *LDLJ* (or related metrics) wherein obtaining stable higher-order derivatives of position is critical for accuracy and interpretability. The Savitzky–Golay (SG) filter is an established and widely used method for derivative estimation from sensor/noisy data. The SG filter demonstrates substantially superior results than discrete finite difference methods for computing higher-order derivatives [Ahnert and Abel (2007)]. Thus, this study explores degrees of SG smoothing for metric computation. Figure 1 illustrates the undesirable effect of noise on the computation of higher-order derivatives motivating the need for this study.

• Are motion smoothness metrics superior to other process metrics in correlating with skill indicator metrics?

In studies seeking to assess clinical skill in a simulator or otherwise, a suite of metrics is typically employed. However, are some metrics more powerful than others in discerning clinical skills? If so, this might have implications for the design of hardware as well as in creating training curricula that involve the most sensitive of metrics. For example, a study on a robotic laparoscopic skill trainer employed a few rudimentary metrics to assess performance [Judkins et al. (2009)] and reported significant differences between the five medical students and five laparoscopic surgeons who participated. In contrast, several studies utilize rudimentary metrics and more complicated motion smoothness metrics to distinguish between skill levels [Gulde and Hermsdörfer (2018b), Kholinne et al. (2018), Belvroy et al. (2020)]. The argument commonly made for using more sophisticated metrics is either that they grasp an aspect of skill not captured by rudimentary metrics or that they do it better. In this study, we examine if any of the process metrics, including both the more sophisticated motion smoothness metrics and the rudimentary metrics, are superior to the other process metrics in correlating with the cannulation skill.

## 4 Materials and Methods

### 4.1 The Cannulation Simulator

**FIGURE 2:**
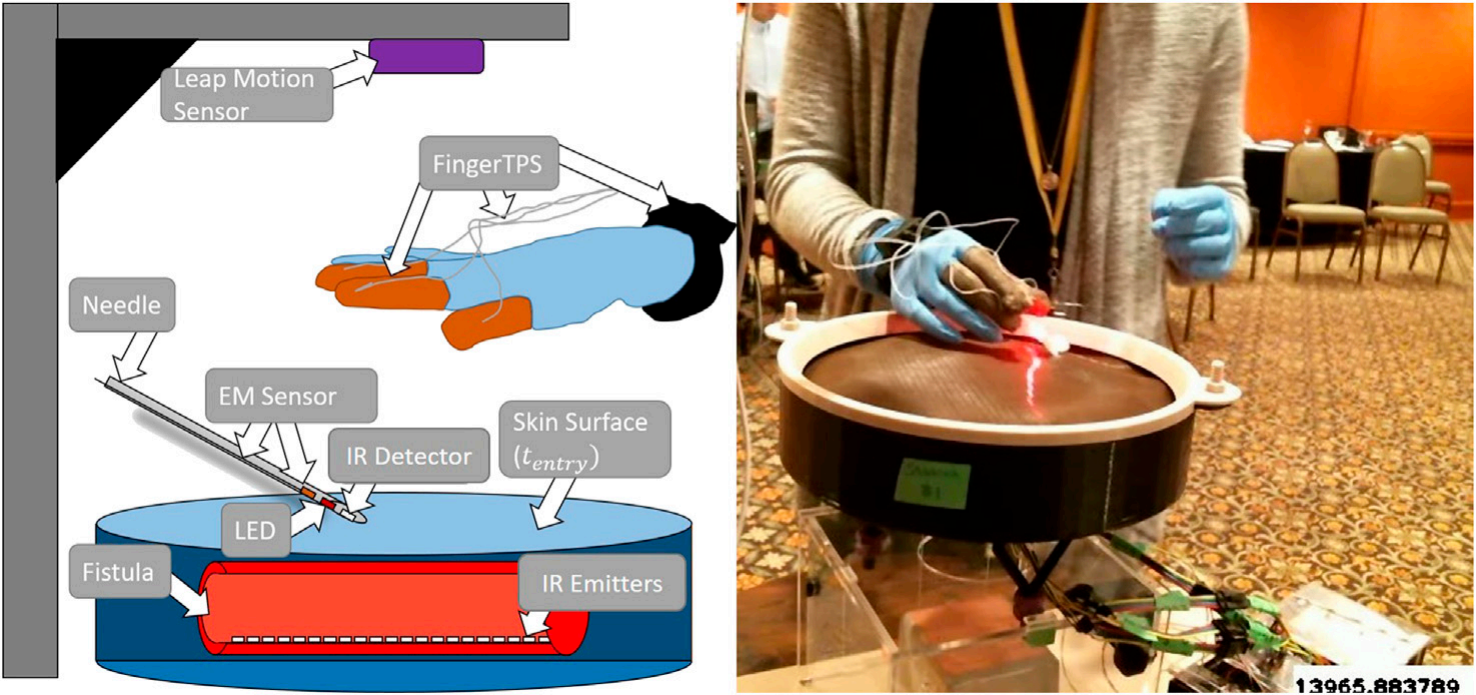
Illustrated sketch of the simulator with a few of the various sensors alongside a physical example of subject receiving flashback. A needle approaches one of the four fistulas. $t_{entry}$ occurs when the needle punctures the fistula.

This study collected and analyzed data from clinicians on a novel simulator for hemodialysis cannulation [Zhang et al. (2019), Liu et al. (2020a), Liu et al. (2020b)]. The simulator comprises four synthetic arteriovenous (AV) fistulas, each outfitted with a vibration motor to simulate turbulent blood flow (termed “thrill” by clinicians) in a fistula. Figure 2 illustrates a sketch of the simulator hardware and the experimental setup. There are four sensors present in the system: an electromagnetic (EM) position sensor (trakSTAR, Northern Digital Inc.) located inside the needle; a force sensing system to record forces applied by the fingers (FingerTPS, Pressure Profile Systems Inc.); the Leap Motion sensor (Ultraleap Inc.) for tracking finger position; and infrared (IR) emitters and detectors for determining whether the needle is inside the fistula. The Leap Motion sensor is affixed above the simulator while the FingerTPS sensors are fit onto the user’s thumb, index, and middle fingers. The IR sensors are embedded within the needle tip and each fistula located in the simulator. An external camera (RealSense, Intel Inc.) records video of participants performing the cannulation task.

Custom software was written in C++ for integrating all sensors to enable data collection at sensor-specific sample rates [Liu et al. (2020b)].

### 4.2 Experimental Design

All participants provided informed consent to participate in the study. Data were recorded from 52 participants comprising of nurses, nurse practitioners, and dialysis technicians ranging from 0 to 38 years of experience. These participants performed four cannulation attempts on each of the four fistulas in the simulator for a total of 16 trials per participant. Data on 53 trials were excluded due to either LED failure, lack of expert rating, failure to complete the trial, or data saving errors, resulting in a total of 779 viable trials for analysis. Before performing the task, each participant filled out a questionnaire that included participants’ cannulation experience. Once the questionnaire was answered, participants were debriefed about the experimental procedure using a self-advanced PowerPoint presentation. Following this, participants were cannulated on the simulator following clinical guidelines as closely as possible. As such, for each attempt, only one (out of the four) fistula’s vibration motor was activated randomly. The subject was instructed to first palpate the skin surface to locate the correct fistula (i.e., the fistula with a “thrill”). They then attempted to insert the needle into the fistula to attempt successful cannulation. If the needle tip successfully entered the fistula, a red LED located inside the cannula was turned on to simulate blood flashback visible in a clinical setting. Following standard guidelines, if a stable blood flashback is procured, the participant is instructed to “level out” (lower the angle of the needle) to allow for taping of the cannula during dialysis.

### 4.3 Data Segmentation

**FIGURE 3:**
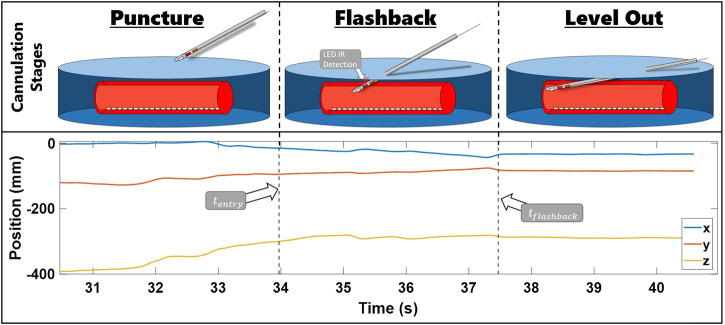
Steps of needle insertion illustrated alongside an example of trial position data. The images show the needle approaching the fistula, entering the fistula and receiving flashback, and leveling out the needle. *x*, *y*, and *z* needle positions during these stages are presented in the bottom row plot. The position data are segmented by $t_{entry}$ (the first dotted line) and $t_{flashback}$ (the second dotted line).

**FIGURE 4:**
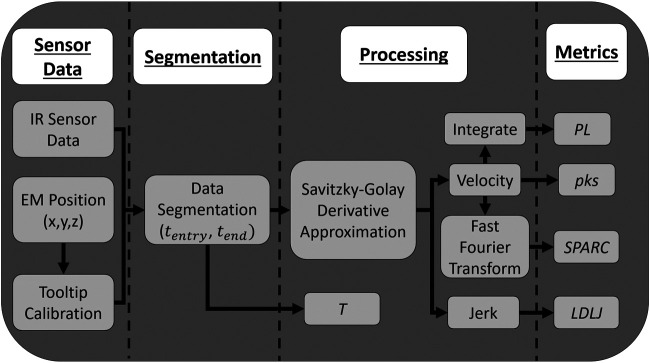
Flowchart presenting how data are collected and processed and how the process metrics are calculated.

Sensor data were collected at a rate of 100 Hz and synchronized in Visual Studio 2017 (Microsoft Inc.), and data segmentation and metric calculations were conducted through MATLAB R2020a (MathWorks Inc.). We computed the position of the needle tip based on the location of the EM sensor inside the needle and needle geometry using a pivot calibration. Following the procedure outlined in Liu et al. (2020b), data were extracted and segmented into specific cannulation subtasks: insertion, flashback, and leveling out (Figure 3). In this figure, a plot of *x*, *y*, and *z* needle positions of a sample cannulation trial is seen. The trial is separated with a dotted line and its subtask is seen in the title. Each subtask also has a corresponding visual sketch above the segmented plot. By comparing the *z*-position of the electromagnetic sensor with the height of the surface of the skin, we determined the timestamp at which the needle punctured the surface of the skin (denoted as $t_{entry}$). Movement data were segmented from $t_{entry}$ until the end of the task ($t_{end}$). This segmentation also allowed for multiple reinsertion attempts that a participant may have used. As has been noted in studies that use motion smoothness metrics, it is essential to constrain the task since motion smoothness metrics are task-dependent. That is, without a consistent start and end point, evaluation of motion smoothness profiles would be meaningless. Once these data are segmented, derivatives are approximated through a third-order (SG) smoothing filter. To compare various degrees of smoothing, SG window spans of minimum (5 samples), a fourth of a second (25 samples), a half of a second (51 samples), 1 s (101 samples), and 2 s (201 samples) were used. A flowchart detailing the data segmentation and the metrics calculation process is shown in Figure 4.

### 4.4 Metrics

#### 4.4.1 Process Metrics

We define dexterity process metrics calculated in our study as follows:

• Time (*T*): The total time from $t_{entry}$ to $t_{end}$ of the task.

$$T=t_{end}-t_{entry}.$$ (1)

• Peaks (*Pks*): The number of local maxima (peaks) in the velocity profile, computed using the built-in MATLAB function.

$$\mathrm{Pks}=findpeaks\left(\frac{dX}{dt}\right),$$ (2)

$$X=\sqrt{x^{2}+y^{2}+z^{2}}.$$ (3)

• Path length (*PL*): The sum of Euclidean distances between points traversed by the needle tip.

$$\mathrm{PL}=\int_{t_{entry}}^{t_{end}}\frac{dX}{dt}$$ (4)

• Log dimensionless jerk (*LDLJ*): The natural log of jerk integrated and squared.

$$\mathrm{LDLJ}=-\ln\left|\frac{T^{5}}{PL^{2}}\int_{t_{entry}}^{t_{end}}{\left(\frac{d^{3}X}{dt^{3}}\right)}^{2}\right|.$$ (5)

• Spectral arc length (*SPARC*): As defined in Balasubramanian et al. (2012), *SPARC* is the arc length of the Fourier transform of the velocity profile, from the provided MATLAB code.

$$\mathrm{SPARC}=-\int_{0}^{\omega_{c}}\left[{\left(\frac{1}{\omega_{c}}\right)}^{2}+{\left(\frac{d\hat{V}\left(\omega \right)}{d\omega }\right)}^{\frac{1}{2}}\right]d\omega ;$$ (6)

$$\hat{V}\left(\omega \right)=\frac{V\left(\omega \right)}{V\left(0\right)}$$ (7)

#### 4.4.2 Skill Indicator Metric Definition

**FIGURE 5:**
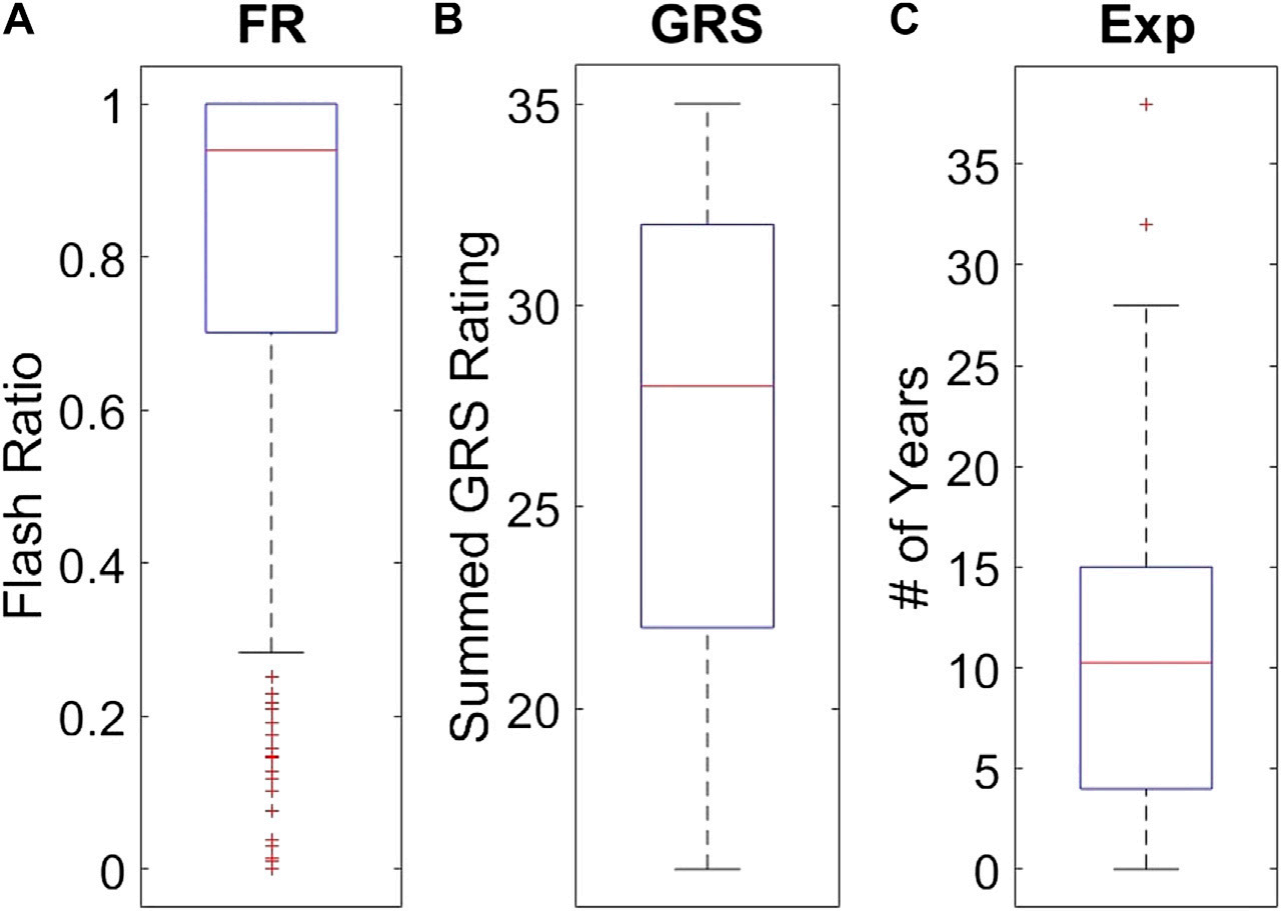
Individual distributions of each skill indicator metric. Subplot **(A)** shows the distribution of **FR** values observed among the 779 trials. Subplot **(B)** shows the distribution of the 49 **GRS** participant values. Subplot **(C)** shows the distribution of 49 **Exp** values for the participants.

For data analysis, we created three statistical models, one for each skill indicator metric, to examine their effectiveness in quantifying skill. A general sense of the descriptive statistics can be gleaned from Figure 5. We defined the three skill indicator metrics as follows:

• Cannulation experience (**Exp**): Subjects were asked to fill out a questionnaire that included the amount of clinical cannulation experience the participant had. Our participants’ years of experience cannulating ranged from 0 to 38 years, with a mean of 11 years and a standard deviation of 8.6 years.
• Global rating sheet (**GRS**): A commonly used method to determine skill level in various medical fields is by experts observing and rating the performance of a task on a Likert-scale questionnaire. Participants were rated by one of three experts on a Likert scale from 1 to 7 on the following aspects: palpation skill, needle holding, needle movement, flashback quality, and overall quality. Ignoring palpation skill, as it was deemed unrelated to the motion of the needle, we summed the scores of the remaining categories for an overall rating for **GRS**. Summed expert scores ranged from 16 to 35, with a mean of 28 and a standard deviation of 6.
• Flash ratio (**FR**): This outcome metric was devised as an objective measure of cannulation task completion. **FR** is defined as the ratio of the sum of total time with flashback to the time from the first flashback Liu et al. (2020b). If flashback stops for any period of time before $t_{end}$, the subject’s score is negatively affected, whereas if no flashback occurs, the subject’s score is 0. It is mathematically expressed as follows:

$$\mathrm{FR}=\frac{\sum \left(t_{flash\_end}-t_{flash\_begin}\right)}{t_{end}-t_{flash}}$$ (8)

Subjects’ scores ranged from 0 to 1, with a mean of 0.79 and a standard deviation of 0.30.

### 4.5 Statistical Analysis

Linear regressions were performed individually for each process metric per each skill indicator metric. Due to the inherent skew of the process metrics, all but *LDLJ* were log-transformed to enable regression modeling. After log transformation, all process metrics were standardized to have zero mean and unit variances to allow for direct comparison of estimated coefficients. We then regressed each skill indicator metric onto each process metric, recording the estimated slopes and associated standard errors. This process was repeated for each window size. After model fitting, pairwise comparisons between estimated coefficients were made. The t-test was chosen for comparing differences between parameters due to ease of interpretation, and since the number of comparisons was large, Tukey’s multiple comparison adjustment was computed for assessing significance at the *α* = 0.05 level based on the equation below. This procedure was repeated comparing association with skill between the various window spans for *LDLJ* and *SPARC* individually.

$$\text{Confidence interval for Tukey’s method}={\bar{\beta }}_{1,i•}-{\bar{\beta }}_{1,j•}\pm \frac{q_{a;r,n-r}}{\sqrt{2}}{\hat{\sigma }}_{\varepsilon }\sqrt{\frac{2}{n}}\;i,j=1,\ldots r;i\neq j.$$ (9)

where *α* corresponds to the significance level 0.05, *β* is the regression coefficient, *q* denotes the critical value of the studentized range distribution, *n* corresponds to the total number of observations (779), *r* is the total number of groups (5 for each analysis), and *i* and *j* are group indicators.

## 5 Results

**FIGURE 6:**
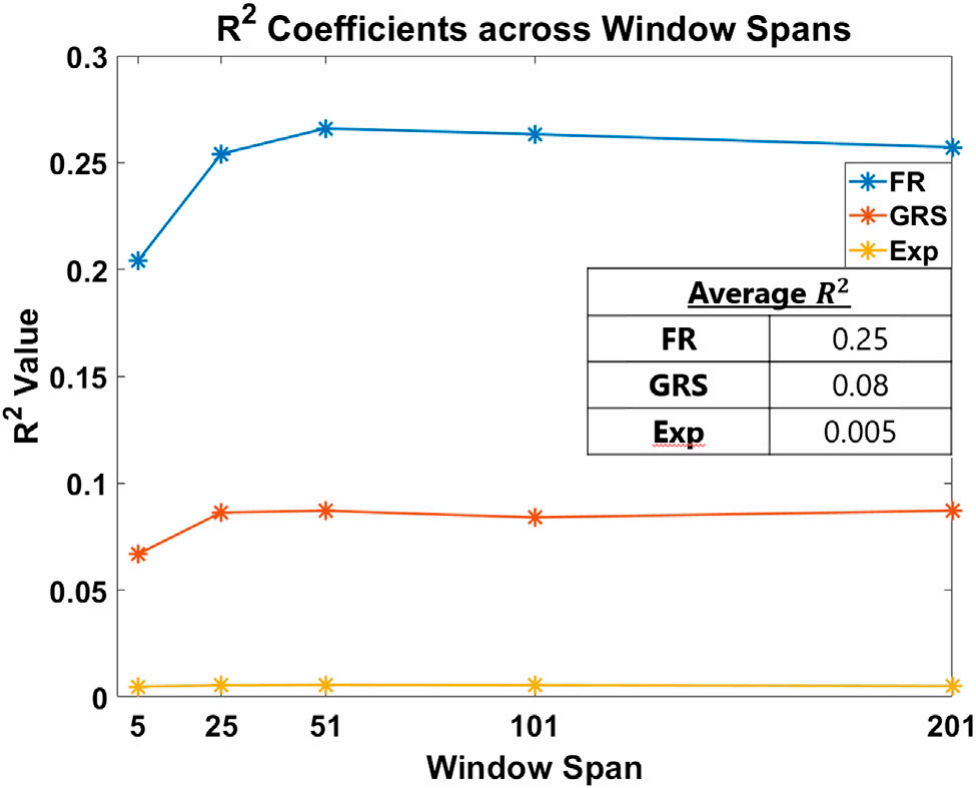
R^2^ coefficient fits of each skill indicator metric are plotted per window span. The averages of these values are presented in the inset table. The objective outcome metric, **FR**, best accounted for the process metrics used in the study to measure skill.

**FIGURE 7:**
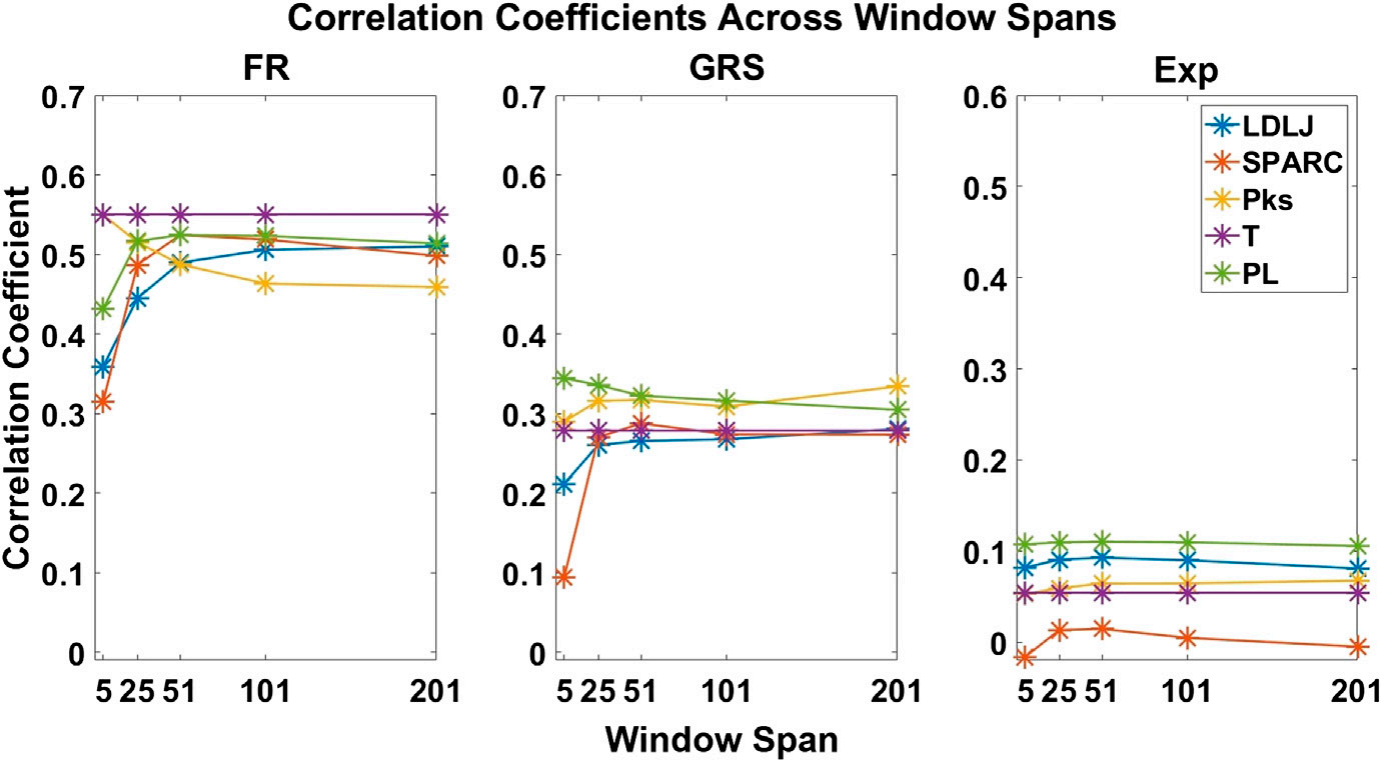
Correlation coefficients of each process metric with respect to each skill indicator metric. The coefficients are plotted across all window spans. This plot demonstrates the effect of the degree of smoothing on the strength of association between process and skill indicator metrics.

The first part of our analysis examined which of the skill indicator metrics best accounted for the dexterity process metrics. We begin by summarizing model fits associated with each regression model. The absolute value of the Pearson correlation coefficient between each process metric and skill indicator metric across all the window sizes and the mean of $R^{2}$ for all regression models are presented in Figure 6. It can be noted that **FR** has a much higher fit than **GRS** (approximately 25 vs. 8%) and that **Exp** has an extremely poor model fit (about 0.5%). It is also worth noting that none of the process metrics are very strongly correlated with **Exp** (as is evidenced in subplot (c) in Figure 7, which demonstrates each process metric’s correlation to the subplot’s indicator metric). Therefore, any conclusions made for **Exp** are not reliable owing to the poor fit. The most salient observation from this analysis is the superiority of **FR**, the objective outcome metric, in accounting for dexterity process metrics.

**FIGURE 8:**
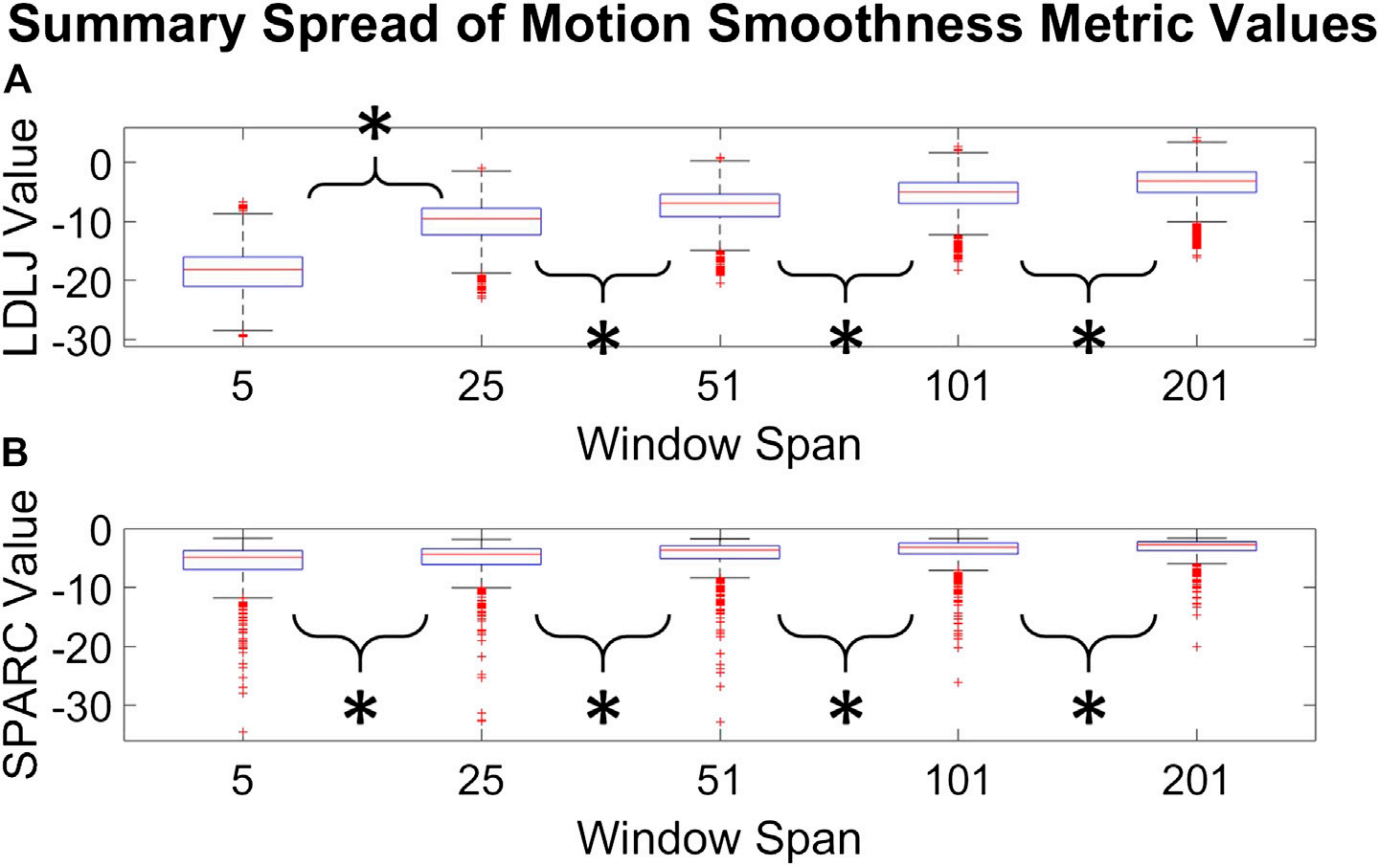
Distributions of *LDLJ* [subplot **(A)**] and *SPARC* [subplot **(B)**] values for each window span. An asterisk indicates that a t-test between two distributions demonstrated statistical significance.

**FIGURE 9:**
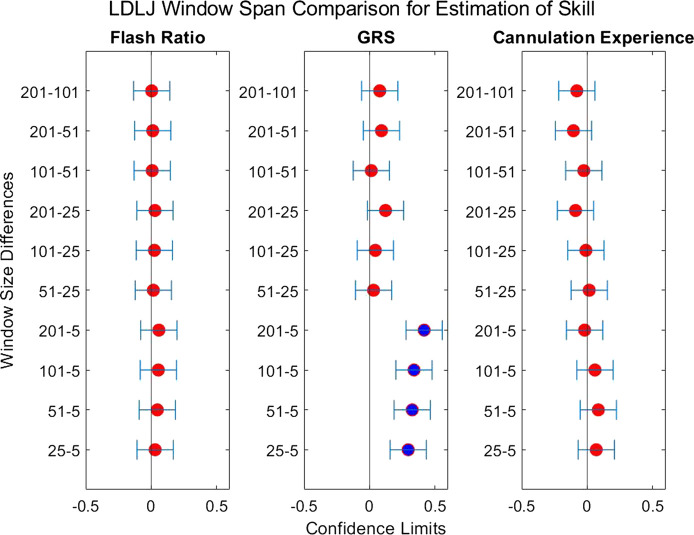
Confidence intervals of each skill indicator metric’s association with *LDLJ* between the tested window spans. If the confidence limits are both positive, the minuend is more associated with the corresponding skill indicator metric, and vice versa if the confidence limits are both negative. If the confidence interval passes through zero, there is no significant difference in the association.

**FIGURE 10:**
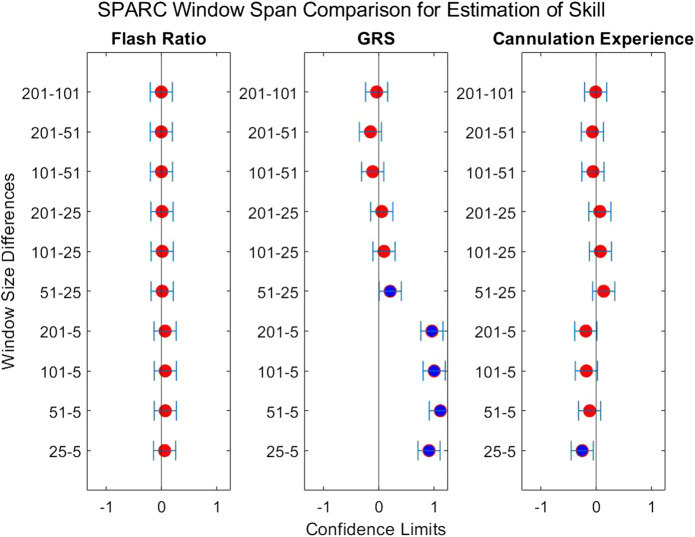
Confidence intervals of each skill indicator metric’s association with *SPARC* between the tested window spans. If the confidence limits are both positive, the minuend is more associated with the corresponding skill indicator metric, and vice versa if the confidence limits are both negative. If the confidence interval passes through zero, there is no significant difference in the association.

The second question we examined in this work is if the degree of smoothing affected the computation of *LDLJ* and *SPARC*, the two motion smoothness metrics used here. Figure 8 reveals the changes in the values of *LDLJ* and *SPARC* when plotted as a function of window span (indicative of the degree of smoothing). This result indicates that motion smoothness values are affected by the degree of smoothing. However, does this change result in a significant difference in the correlation of *LDLJ* or *SPARC* with each skill indicator metric? This question is important in the context of this study which seeks to investigate the power of metrics to quantify skill. As seen in Figures 9, 10, the window size does not affect the correlation of *LDLJ* and *SPARC* with **FR**. Even the “noisiest” span of five correlated well with **FR** and the other spans. For **GRS**, however, a window span of five is significantly less associated with **GRS** than the other window spans for both *LDLJ* and *SPARC*. That is, when minimal smoothing is applied, metric values do not correlate with **GRS** as well.

**FIGURE 11:**
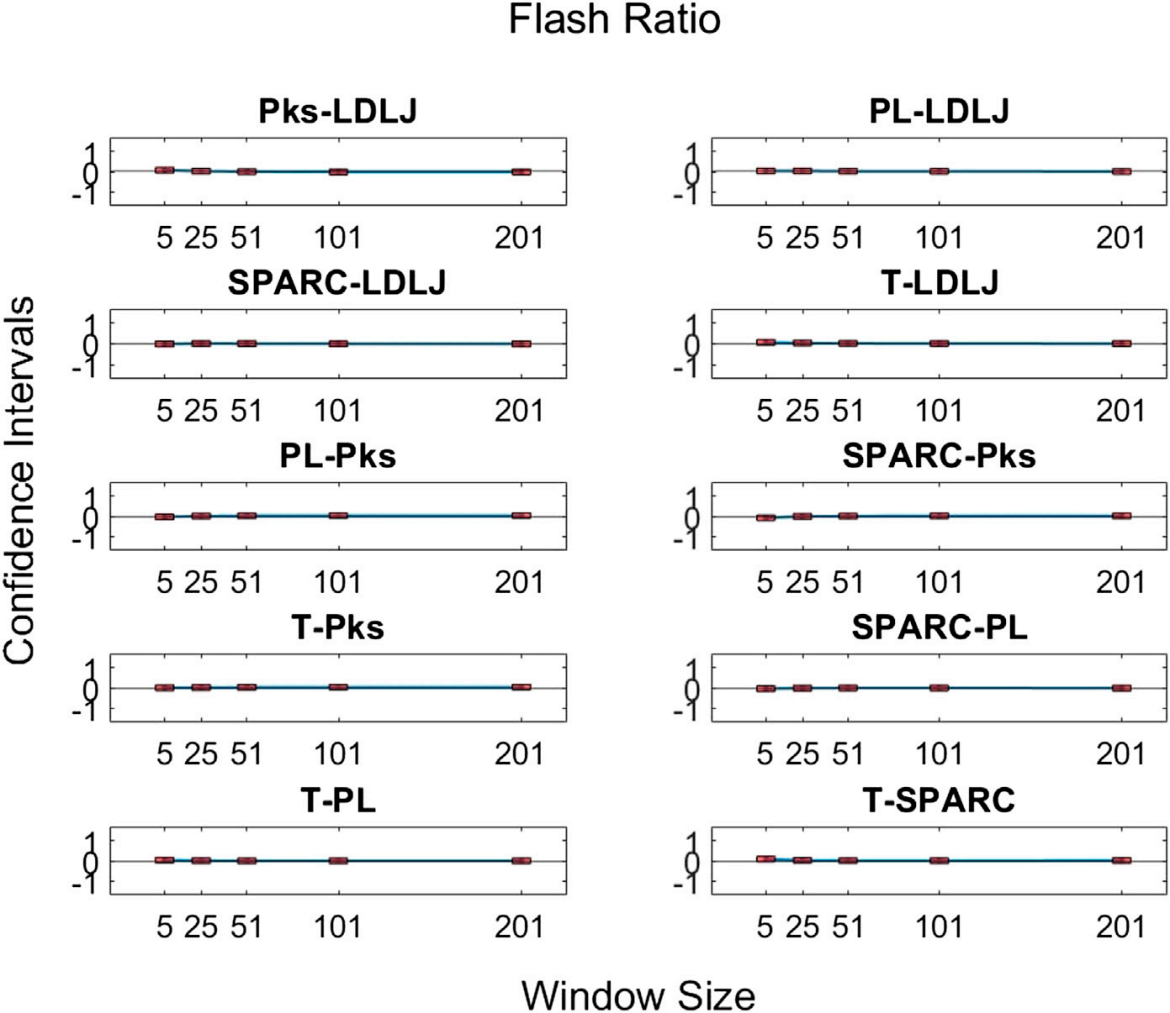
Confidence intervals of pairwise comparisons of process metrics with respect to **FR** plotted against all tested window spans. To visualize the relationships between effects sizes of process metrics and window spans, a line is plotted through the point estimates. A horizontal line is drawn through 0. If the confidence limits are both above zero, the minuend is more strongly associated with **FR**; if the limits pass through zero, the differences are insignificant; and if the limits are below zero, the subtrahend is more strongly associated with **FR**.

**FIGURE 12:**
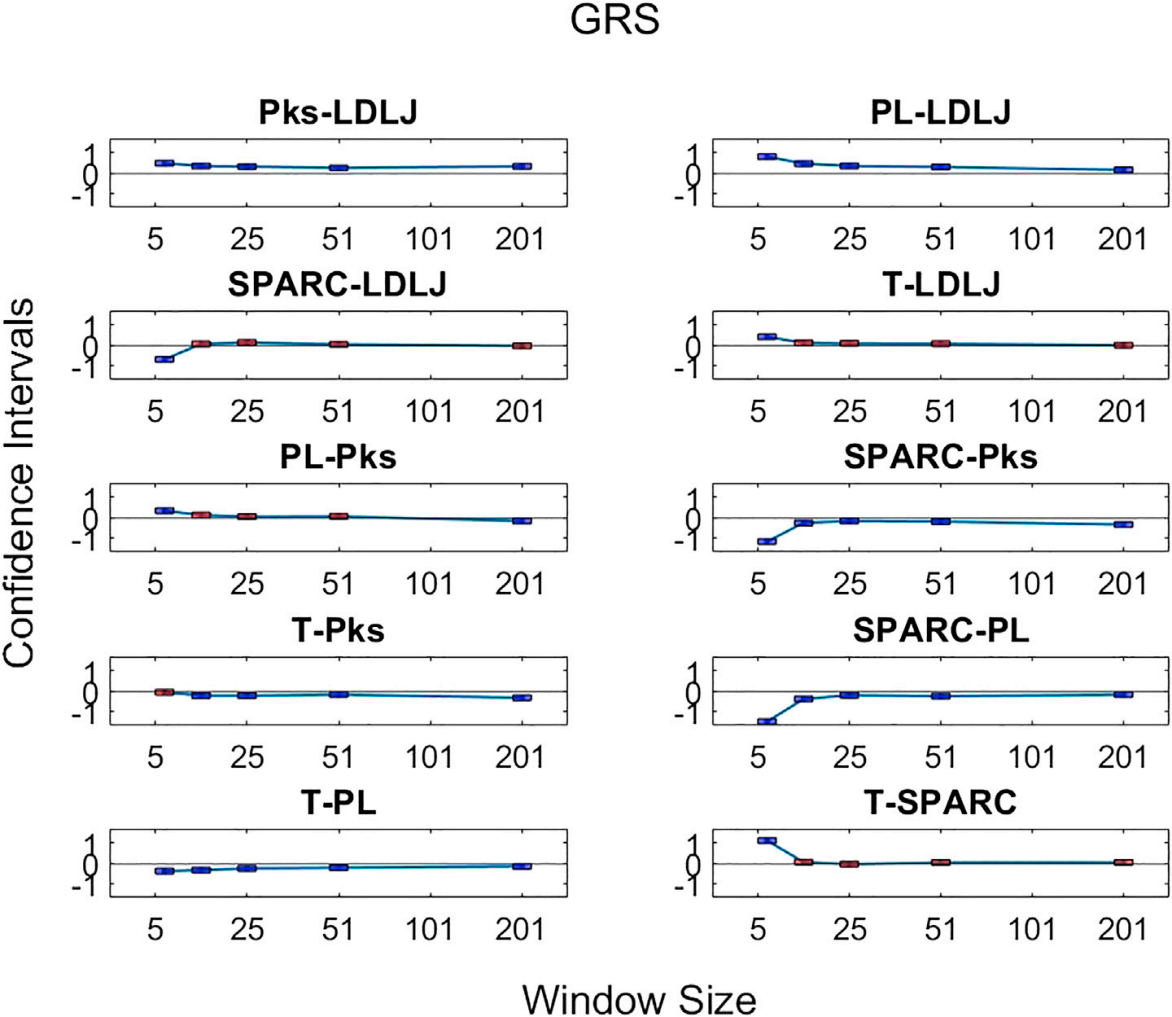
Confidence intervals of significant differences of pairwise comparisons of process metrics with respect to **GRS** plotted against all tested window spans. A line is drawn through the intercepts to better visualize the trend of the confidence intervals of each window span increase. If the confidence interval is above zero, the minuend is more strongly associated with **GRS**; if it passes through zero, the differences are insignificant; and if it is below zero, the subtrahend is more strongly associated with **GRS**.

**FIGURE 13:**
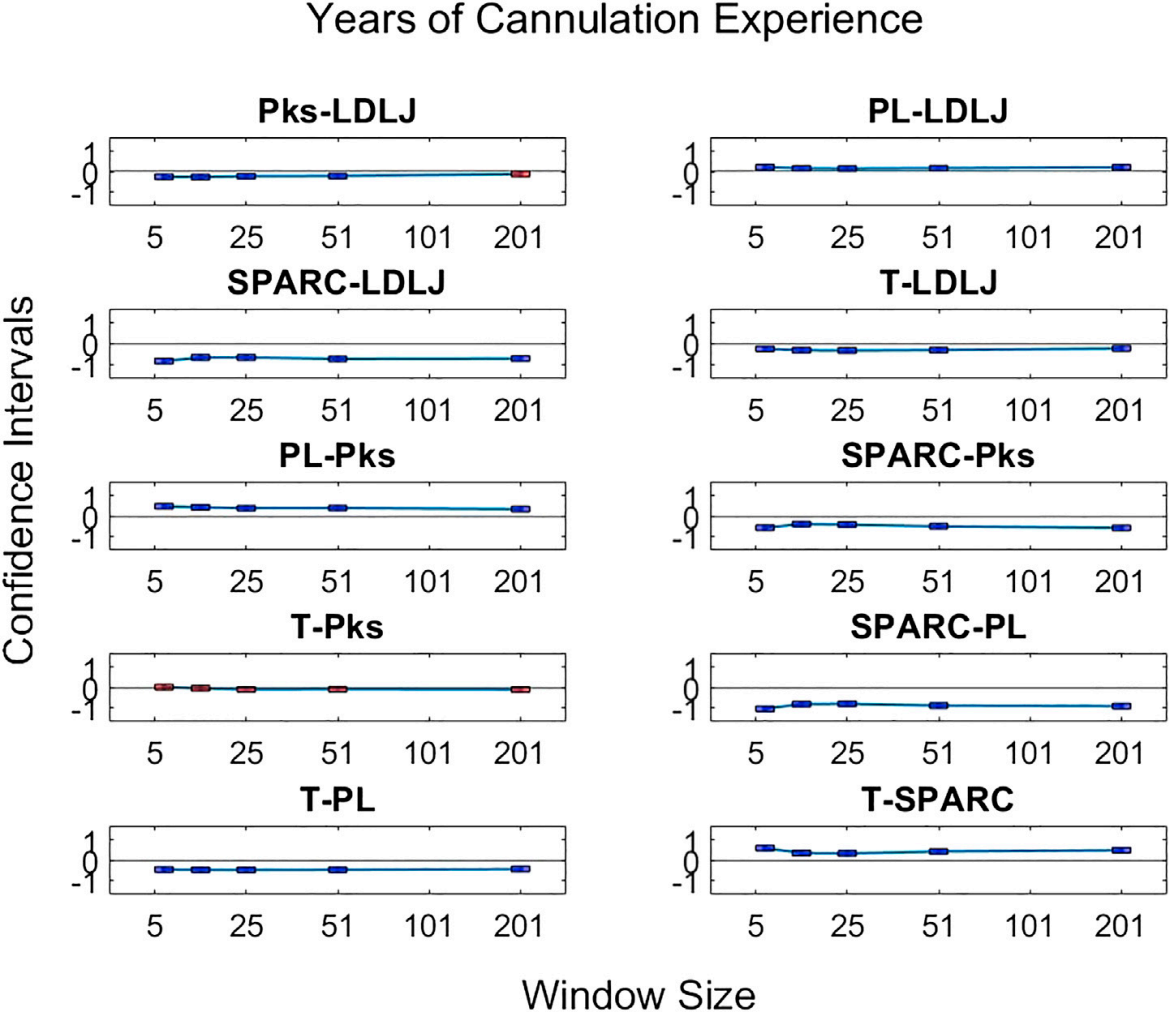
Confidence intervals of significant differences of pairwise comparisons of process metrics with respect to **Exp** plotted against all tested window spans. A line is drawn through the intercepts to better visualize the trend of the confidence intervals of each window span increase. If the confidence interval is above zero, the minuend is more strongly associated with **Exp**; if it passes through zero, the differences are insignificant; and if it is below zero, the subtrahend is more strongly associated with **Exp**.

The third research question we examined is if motion smoothness metrics are superior to other process metrics in correlating with skill indicator metrics. Figures 11–13 show the confidence intervals of the significant differences of association with skill among the process metrics across the five window spans for each skill indicator metric. It is important to note that the formulations of *PL*, *T*, and *Pks* have a negative modifier added to the value to enable comparisons between the slopes. As a result, a decrease in any of the process metric values denotes a worse performance. Our pairwise comparison results demonstrate that no process metric is significantly better than any other metric in association with **FR**. This holds true across all window spans. While some significant differences were observed in association with **GRS**, we conclude that these differences are erratic, since neither of the process metrics demonstrates consistency in superior association with the other process metrics across window spans. Note from the earlier discussion that **GRS** has a low $R^{2}$ of about 8%. *PL* indicates a consistently higher association with **Exp** in comparison with the other metrics. However, due to the model’s exceptionally poor fit, it is difficult to make any meaningful assertions regarding **Exp**.

## 6 Discussion

### 6.1 Which Skill Indicator Metric Best Accounts for Dexterity Measures?

Methods for classifying the level of skill must be robust for effective application in medical training simulators. In this study, we present an objective outcome metric for assessing the degree of success in a simulated clinical task and examined its power for quantifying skill in comparison with the more generally used skill indicator metrics. In addition, unlike many studies that binarize expertise into “expert” or “novice,” we collected years of clinical experience as a finer-grained measure for analysis. This detail in our experimental design enabled the investigation of the research questions presented in this work. From our results, the objective outcome metric **FR** better accounted for the process metrics used in the study to capture skilled movement. Traditionally used metrics as surrogates for clinical skill demonstrated inferior performance to quantify dexterity. Furthermore, the correlation coefficients for each process metric in Figure 7 drastically changed in association with each skill indicator metric, with **FR** having the best correlations.

In Figure 6, the overall fit for each indicator metric, although comparatively different, is relatively low (about 25% for **FR**). This can be attributed to the process metrics examined accounting primarily for dexterity. We would likely see improvements in model fit if other aspects of skill, such as force, needle positioning and angle, and decision making, are measured and incorporated. Despite being a regularly used skill indicator metric in the field of medical skills training, **Exp** performs poorly for the cannulation task, accounting for only about 0.5% of the variation in the model. It is important to take into account the current task: although cannulation is an important medical procedure, it is not as complicated and multifaceted as surgery. As a result, it is possible that simply having experience in cannulation does not necessarily result in increased skills for successful cannulation. We can surmise that **Exp** is not a useful measure of skill in our cannulation simulator.

**GRS** yields a better fit than **Exp**, but still relatively poor at about 8.2%. This increase is likely due to the expert's knowledge of skill as he or she rates canulation performance by direct observation. This metric, therefore, provides a better assessment of skill than **Exp**. Nevertheless, the fit is still relatively small and can be attributed to two primary reasons: (1) **GRS** evaluates each subject as a whole, rather than on a trial-by-trial basis, and (2) expert raters lack true knowledge of the success of a task from mere observation. In contrast, **FR** measures the true success of the trial and, consequently, sees a significant improvement in model fit at about 25%. This result may encourage the formulation of more outcome metrics to measure the degree of success in a clinical task objectively. Some examples of this are measuring the leakage after a surgeon sutures a vascular anastomosis or measuring the degree of motion after orthopedic surgery. Such metrics may yield a truer measure of skill, potentially impacting skill assessment and training in a positive way.

### 6.2 Does the Degree of Smoothing Significantly Affect the Computation of Motion Smoothness Metrics?

As mentioned earlier, there has been some discussion in recent literature on the relative benefits of *SPARC* and *LDLJ* in measuring motion smoothness. One limitation pointed out is *LDLJ’s* sensitivity to noise due to its use of the third derivative of position. In comparison, *SPARC* uses velocity, requiring only one derivative of position with respect to time. The effects of derivative computation and sensor data smoothing have not been systematically explored in the literature. We hypothesized that the degree of smoothing affects the computation of both motion smoothness metrics. As seen in Figure 8, both *LDLJ* and *SPARC* see significant differences in means as a function of window span. Nevertheless, as also evidenced in our results, the significant differences of means between window spans did not affect the discerning power of motion smoothness metrics in our task, calculated using correlation coefficients and regression slope comparisons across a variety of SG window spans. In comparison with the other process metrics, *LDLJ* and *SPARC* see the largest increase in correlation when moving from a window span of 5 to 25 for each skill indicator metric in Figure 7. This is most likely due to the noise present in sensor data not being adequately filtered in the small window span. After the window span of 25 (some smoothing), the stability of *LDLJ* and *SPARC* is similar.

When comparing the effect of the degree of smoothing (*via* window spans) on the strength of correlation between indicator metrics and motion smoothness metrics (Figures 9, 10), we see no significant differences in association with **FR** for both *LDLJ* and *SPARC* across all window spans. As a result, the level of smoothing does not ultimately affect the motion smoothness metrics’ level of association with the objective outcome metric in our study. On the other hand, a window span of five is significantly less associated with **GRS** than all other window spans for both motion smoothness metrics. Most of these differences become insignificant as the degree of smoothing increases. This is likely due to the noise present in the data at window span five, causing motion smoothness metric values to be more erratic. These results follow the trend that a moderate amount of smoothing, particularly when calculating derivatives, is important for more robust results. We refrain from making any claims for **Exp** since this skill indicator metric has a poor model fit.

There are few studies examining derivative formulations and smoothing for motion smoothness calculations present in the literature. An in-depth study on the effects of noise on various motion smoothness metrics raises concern with *LDLJ’s* sensitivity to noise, though it was deemed not as sensitive as *Pks* or *DLJ* [Balasubramanian et al. (2012)]. In contrast, Balasubramanian et al. (2012) presented *SPARC* as a viable metric due to its robustness to noise. However, motion smoothness metrics are task-dependent; a recent study reported that *SPARC* may not be as effective as *LDLJ* in a specific application [Melendez-Calderon et al. (2020)]. The choice of motion smoothness metrics used in a study should depend on the task performed. Our work also brings to light the importance of the quality of smoothing. Gulde and Hermsdörfer (2018a) reported that minimal window spans yielded the largest relative deviations, whereas window spans between 280 and 690 ms had the lowest relative deviations. One limitation of this study is that the effect of derivative calculations was not examined since jerk-based motion smoothness metrics were not computed. To examine the effects of smoothing parameters on derivative approximations, we chose the SG method due to its superiority over finite difference methods [Ahnert and Abel (2007)]. We saw no significant differences in association with skill after a reasonable degree of smoothing for *LDLJ* with the highest fit skill indicator metric, **FR**. Similar to Gulde and colleagues’ findings, for *LDLJ* and *SPARC*, the window span of five had significantly less association with **GRS** than the other window spans. Consequently, a reasonable level of smoothing enables meaningful use of motion smoothness metrics for skill assessment.

### 6.3 Are Motion-Based Process Metrics Superior in Correlation to Skill?

When *LDLJ* and *SPARC* are used alongside other metrics like *T* and *PL* in the literature, there is a lack of direct comparison between the relative powers of these metrics to discern skill. For example, *PL* was unable to demonstrate a significant difference between novices and experts, while both *SPARC* and idle time distinguished between the groups [Belvroy et al. (2020)]. One reason for this may be that *PL* may not effectively quantify surgical skill in the FEVS simulator. Even so, is there a benefit to using one metric over another (e.g., *SPARC* over idle time) if they can both determine significant differences in the groups? Similarly, when comparing hand movements of elderly vs. young patients, though all metrics demonstrated significance, *Pks* normalized per meter demonstrated a higher multiple linear regression R-squared than *SPARC* [Gulde and Hermsdörfer (2018b)]. However, in another study on the FEVS, *Pks* did not have a significant correlation to skill, whereas *DLJ* and *SPARC* did, with *SPARC* having the higher correlation to skill out of the two [Estrada et al. (2016)]. In our results, *T* had a higher correlation to **FR**, as expected due to the nature of how **FR** is formulated. Nonetheless, *PL* had similar or higher correlations when compared to *SPARC* and *LDLJ*.

By directly comparing how the process metrics associate with skill, we desired to determine if any process metric was more significantly superior in association with the skill to each other due to their varying results. As observed in Figure 11, there are no significant differences in the pairwise comparisons of the strength of the process metrics’ association with **FR**. Note that **FR** has the highest reliability for assessing skill in the study. In contrast, significant differences in association with **GRS** were observed in some cases in Figure 12. The differences were generally seen when the degree of smoothing was minimal (window span = 5). One may infer from this that, after some smoothing is applied to position sensor data, both motion smoothness metrics may have a similar ability to discern skill. This result is reiterated by noting the correlation plots in Figure 7. Correlation coefficients are the least at the lowest window span with the only minimal smoothing (and the greatest amount of potential noise). After some smoothing, however, the correlation coefficients become relatively stable.

Based on our results, we conclude that having a strong measure of skill like the objective outcome metric **FR** yielded the most stable set of process metric comparisons. **GRS**, on the other hand, yielded less explainable results as a function of metrics and degree of smoothing. One key finding of this work is the need for a robust amount of smoothing and an accurate metric for measuring skill outcome.

Our study does not test whether each process metric can predict the value of each skill indicator metric, but rather each process metric’s association with that skill indicator metric. Future work involving said prediction models could provide insight into the superiority of the process metrics. It is also important to take into account the dependence of task constraint of motion smoothness metrics when making these analyses: the cannulation procedure consists of a simple motion from $t_{entry}$ to $t_{end}$. The task may not be complex enough to see these differences in association with skill. Another aspect we wish to highlight is that the efficacy and superiority of motion smoothness metrics depend on the task being studied. As mentioned previously, motion smoothness metrics capture the dexterity of hand movements, examining precise movements. Our cannulation simulator task does not require precise motion, possibly causing the lack of significant difference in process metric association with **FR**. Previous studies report varying results on process metrics differentiating between experts and novices, yet motion smoothness metrics tend to consistently demonstrate significant differences if effective calculations are performed. Therefore, despite their task dependency, we can conclude that motion smoothness metrics are at least as effective in their skill evaluation as other process metrics. Future work on a simulator involving testing across tasks requiring different hand movements would allow for a study on the robustness of motion smoothness metrics vs. other process metrics.

### 6.4 Conclusion

Clinical skills training is critical for sustaining an efficient workforce. The use of remote and automated simulators for skills training is especially appealing. In this study, we demonstrate that commonly used skill indicator metrics may be limited in their assessment. In contrast, an objective metric for measuring the degree of task success proved to be a superior skill indicator metric, exhibiting stronger goodness of fit to process metrics. Moreover, the lack of significant differences in association in the process to **FR**, combined with the much higher model fit, may demonstrate the robustness of this measure. Our results also demonstrated that the degree of smoothing of sensor data affects the computation of motion smoothness metrics under certain conditions. These results directly inform the design and use of simulator-based training methods. The flexibility of training simulators is an excellent asset towards effective training of medical practitioners and students, but it is vital to optimize simulators for efficient use in remote, automated assessment without the need for expert raters or on-site training.

## Data Availability

The datasets presented in this article are not readily available because of restrictions on data access placed by the relevant IRB. Inquiries may be directed to joseph@clemson.edu.

## References

[B1] Ahnert K. Abel M. (2007). Numerical differentiation of experimental data: local versus global methods. Comp. Phys. Commun. 177, 764–774. 10.1016/j.cpc.2007.03.009

[B2] Balasubramanian S. Melendez-Calderon A. Burdet E. (2012). A robust and sensitive metric for quantifying movement smoothness. IEEE Trans. Biomed. Eng. 59, 2126–2136. 10.1109/TBME.2011.2179545 22180502

[B3] Balasubramanian S. Melendez-Calderon A. Roby-Brami A. Burdet E. (2015). On the analysis of movement smoothness. J. NeuroEng. Rehabil. 12, 112. 10.1186/s12984-015-0090-9 26651329 PMC4674971

[B4] Belvroy V. M. Murali B. Sheahan M. G. O’Malley M. K. Bismuth J. (2020). Motion metrics reliably differentiate competency: Fundamentals of endovascular and vascular surgery. J. Vasc. Surg. 72, 2161. 10.1016/j.jvs.2020.02.047 32276027

[B5] Braman J. P. Sweet R. M. Hananel D. M. Ludewig P. M. Van Heest A. E. (2015). Development and validation of a basic arthroscopy skills simulator. Arthroscopy 31, 104–112. 10.1016/j.arthro.2014.07.012 25239171

[B6] Brouwer D. J. (2011). Cannulation camp: basic needle cannulation training for dialysis staff. Dial. Transpl. 40, 434–439. 10.1002/dat.20622

[B7] Duran C. Estrada S. O’Malley M. Sheahan M. G. Shames M. L. Lee J. T. (2015). The model for Fundamentals of endovascular surgery (fevs) successfully defines the competent endovascular surgeon. J. Vasc. Surg. 62, 1660–1666. 10.1016/j.jvs.2015.09.026 26598123

[B8] Estrada S. Duran C. Schulz D. Bismuth J. Byrne M. D. O’Malley M. K. (2016). Smoothness of surgical tool tip motion correlates to skill in endovascular tasks. IEEE Trans. Human-Mach. Syst. 46, 647–659. 10.1109/THMS.2016.2545247

[B9] Farnworth L. R. Lemay D. E. Wooldridge T. Mabrey J. D. Blaschak M. DeCoster T. A. (2001). A comparison of operative times in arthroscopic ACL reconstruction between orthopaedic faculty and residents: the financial impact of orthopaedic surgical training in the operating room. Iowa Orthop. J. 21, 31–35. 11813948 PMC1888196

[B10] Flash T. Hogan N. (1985). The coordination of arm movements: an experimentally confirmed mathematical model. J. Neurosci. 5, 1688–1703. 10.1523/JNEUROSCI.05-07-01688.1985 4020415 PMC6565116

[B11] Ghasemloonia A. Maddahi Y. Zareinia K. Lama S. Dort J. C. Sutherland G. R. (2017). Surgical skill assessment using motion quality and smoothness. J. Surg. Edu. 74, 295–305. 10.1016/j.jsurg.2016.10.006 27789192

[B12] Goldvasser D. McGibbon C. A. Krebs D. E. (2001). High curvature and jerk analyses of arm ataxia. Biol. Cybern. 84, 85–90. 10.1007/s004220000201 11205353

[B13] Goyal S. Radi M. A. Ramadan I. K.-A. Said H. G. (2016). Arthroscopic skills assessment and use of box model for training in arthroscopic surgery using sawbones – “FAST” workstation. SICOT-J. 2, 37. 10.1051/sicotj/2016024 27801643 PMC5089855

[B14] Gulde P. Hermsdörfer J. (2018a). “A comparison of smoothing and filtering approaches using simulated kinematic data of human movements,” in Proceedings of the 11th International Symposium on Computer Science in Sport (IACSS 2017), Editors. Lames M. Saupe D. Wiemeyer J. (Cham, Switzerland: Springer International Publishing), 663. 97–102. 10.1007/978-3-319-67846-7-10

[B15] Gulde P. Hermsdörfer J. (2018b). Smoothness metrics in complex movement tasks. Front. Neurol. 9. 615. 10.3389/fneur.2018.00615 30258393 PMC6143727

[B16] Hafford M. L. Van Sickle K. R. Willis R. E. Wilson T. D. Gugliuzza K. Brown K. M. (2013). Ensuring competency: are Fundamentals of laparoscopic surgery training and certification necessary for practicing surgeons and operating room personnel? Surg. Endosc. 27, 118–126. 10.1007/s00464-012-2437-7 22773236

[B17] Hogan N. Sternad D. (2009). Sensitivity of smoothness measures to movement duration, amplitude and arrests. J. Mot. Behav. 41, 529–534. 10.3200/35-09-004-RC 19892658 PMC3470860

[B18] Hung A. J. Oh P. J. Chen J. Ghodoussipour S. Lane C. Jarc A. (2019). Experts vs super-experts: differences in automated performance metrics and clinical outcomes for robot-assisted radical prostatectomy. BJU Int. 123, 861–868. 10.1111/bju.14599 30358042

[B19] Jacobsen M. E. Andersen M. J. Hansen C. O. Konge L. (2015). Testing basic competency in knee arthroscopy using a virtual reality simulator: exploring validity and reliability. J. Bone Joint Surg. Am. 97, 775–781. 10.2106/JBJS.N.00747 25948525

[B20] Judkins T. N. Oleynikov D. Stergiou N. (2009). Objective evaluation of expert and novice performance during robotic surgical training tasks. Surg. Endosc. 23, 590–597. 10.1007/s00464-008-9933-9 18443870

[B21] Kholinne E. Gandhi M. J. Adikrishna A. Hong H. Kim H. Hong J. (2018). The dimensionless squared jerk: an objective parameter that improves assessment of hand motion analysis during simulated shoulder arthroscopy. Biomed. Res. Int. 2018, 1–8. 10.1155/2018/7816160 PMC6076914 30105247

[B22] Liu Z. Bible J. Wells J. Vadivalagan D. Singapogu R. (2020a). Examining the effect of haptic factors for vascular palpation skill assessment using an affordable simulator. IEEE Open J. Eng. Med. Biol. 1, 228–234. 10.1109/OJEMB.2020.3017156 33681817 PMC7932134

[B23] Liu Z. Petersen L. Zhang Z. Singapogu R. (2020b). “A method for segmenting the process of needle insertion during simulated cannulation using sensor data,” in 2020 42nd Annual International Conference of the IEEE Engineering in Medicine & Biology Society (EMBC), (Montreal, QC: IEEE), 6090–6094. 10.1109/EMBC44109.2020.9176158 33019360

[B24] Liu Z. Zhang Z. Kunkel D. Roy-Chaudhury P. Singapogu R. (2021). Is experience in hemodialysis cannulation related to expertise? A metrics-based investigation for skills assessment. Ann. Biomed. Eng. 10.1007/s10439-020-02708-5 PMC8263797 33417054

[B25] MacDonald J. Williams R. G. Rogers D. A. (2003). Self-assessment in simulation-based surgical skills training. Am. J. Surg. 185, 319–322. 10.1016/S0002-9610(02)01420-4 12657382

[B26] Melendez-Calderon A. Shirota C. Balasubramanian S. (2020). Estimating movement smoothness from inertial measurement units. Front. Bioeng. Biotechnol. 8, 558771. 10.1101/2020.04.30.069930 33520949 PMC7841375

[B27] O’Malley M. K. Byrne M. D. Estrada S. Duran C. Schulz D. Bismuth J. (2019). Expert surgeons can smoothly control robotic tools with a discrete control interface. IEEE Trans. Hum. Mach. Syst. 49, 388–394. 10.1109/THMS.2019.2919744

[B28] Pedowitz R. A. Esch J. Snyder S. (2002). Evaluation of a virtual reality simulator for arthroscopy skills development. Arthroscopy 18, 1–6. 10.1053/jars.2002.33791 12098111

[B29] Rohrer B. Fasoli S. Krebs H. I. Hughes R. Volpe B. Frontera W. R. (2002). Movement smoothness changes during stroke recovery. J. Neurosci. 22, 8297–8304. 10.1523/JNEUROSCI.22-18-08297.2002 12223584 PMC6758113

[B30] Rosen K. R. (2008). The history of medical simulation. J. Crit. Care 23, 157–166. 10.1016/j.jcrc.2007.12.004 18538206

[B31] Smith M. A. Brandt J. Shadmehr R. (2000). Motor disorder in huntington’s disease begins as a dysfunction in error feedback control. Nature 403, 544–549. 10.1038/35000576 10676962 PMC2556233

[B32] Teulings H.-L. Contreras-Vidal J. L. Stelmach G. E. Adler C. H. (1997). Parkinsonism reduces coordination of fingers, wrist, and arm in fine motor control. Exp. Neurol. 146, 159–170. 10.1006/exnr.1997.6507 9225749

[B33] Van Loon M. M. Kessels A. G. H. Van Der Sande F. M. Tordoir J. H. M. (2009). Cannulation and vascular access-related complications in hemodialysis: factors determining successful cannulation. Hemodial. Int. 13, 498–504. 10.1111/j.1542-4758.2009.00382.x 19840142

[B34] Wininger M. Kim N.-H. Craelius W. (2009). Spatial resolution of spontaneous accelerations in reaching tasks. J. Biomech. 42, 29–34. 10.1016/j.jbiomech.2008.10.015 19062017

[B35] Zhang Z. Liu Z. Singapogu R. (2019). Extracting subtask-specific metrics toward objective assessment of needle insertion skill for hemodialysis cannulation. J. Med. Robot. Res. 4, 1942006. 10.1142/S2424905X19420066 33681506 PMC7932179

